# Genetic and Environmental Factors Shaping Hearing Loss: Xenobiotics, Mechanisms and Translational Perspectives

**DOI:** 10.3390/jox16010027

**Published:** 2026-02-05

**Authors:** Francisco Esteves, Helena Caria

**Affiliations:** 1 Instituto Politécnico de Setúbal (IPS), Escola Superior de Saúde (ESS), Departamento de Ciências Biomédicas, Estefanilha, 2910-761 Setúbal, Portugal; helena.caria@ess.ips.pt; 2 Comprehensive Health Research Centre (CHRC), NOVA Medical School|Faculty of Medical Sciences, Universidade NOVA de Lisboa, 1169-056 Lisboa, Portugal; 3 Biosystems and Integrative Sciences Institute (BioISI), Faculdade de Ciências, Universidade de Lisboa, 1749-016 Lisbon, Portugal

**Keywords:** hearing loss, deafness associated genes, gene-environment interactions, genetic susceptibility, cochlear degeneration, oxidative stress, environmental pollutants

## Abstract

The central mechanistic hypothesis underlying multifactorial hearing loss posits that genetic susceptibility and environmental exposures act synergistically to disrupt cochlear homeostasis through redox imbalance, mitochondrial dysfunction, and pro-inflammatory mechanisms. This gene–environment paradigm has significant translational implications: elucidating the molecular crosstalk between genetic variants and environmental factors may enable precision risk stratification and the development of targeted otoprotective strategies. The present review provides a comprehensive examination of the major determinants implicated in hearing loss. The manuscript is organized into six main sections that encompass the most relevant domains of current research. First, it offers (I) an overview of epidemiological patterns and the multifactorial nature of hearing impairment. This is followed by (II) a background synthesis of the complex genetic architecture underlying hearing loss. Next, the authors present (III) an outline of environmental determinants and exposure profiles associated with auditory dysfunction, highlighting prominent pollutant/xenobiotic classes (e.g., organic solvents and volatile aromatic hydrocarbons, heavy metals, pesticides, and especially organophosphates and persistent organochlorine compounds), followed by (IV) an analysis of oxidative stress, mitochondrial impairment, and inflammatory pathways involved in cochlear injury. Subsequently, (V) translational perspectives and integrated therapeutic approaches are discussed, with emphasis on epidemiological prevention and precision-based interventions. Finally, (VI) this review addresses current challenges and future directions in elucidating gene–environment interactions in hearing loss.

## 1. Introduction and Background

### 1.1. Epidemiological Perspective

Hearing loss stands among the most widespread sensory disorders globally, posing a pressing public-health challenge with profound socioeconomic and cognitive consequences throughout life. According to recent data from the World Health Organization (WHO), more than 1.5 billion people (nearly one in five globally) live with some degree of hearing loss, and about 430 million require rehabilitation for disabling impairment [1,2]. The global burden continues to increase in parallel with population aging, urbanization, and industrialization, reflecting the convergence of genetic predisposition, environmental exposure, and lifestyle-related determinants [3,4,5,6,7,8,9,10]. This trend positions hearing loss among the leading chronic conditions of the twenty-first century and underscores significant regional inequities, as the majority of severe cases occur in low- and middle-income countries, where audiological services and environmental safety measures remain limited [11,12,13,14,15,16,17,18].

Hearing impairment represents a hallmark example of a multifactorial disease, where both endogenous and exogenous contributors converge to determine individual susceptibility and progression [19,20]. Notably, the genetic etiology of hearing loss is highly relevant, as advances in understanding genetic causes have led to significant improvements in quality of life worldwide [21]. Beyond genetic and age-related etiologies, a growing body of epidemiological and mechanistic evidence demonstrates that environmental and occupational pollutants—particularly ototoxic xenobiotics—substantially contribute to both the onset and progression of hearing loss [22,23,24]. Continuous exposure to volatile organic solvents (benzene, toluene, xylene, styrene), heavy metals such as lead and cadmium, and agricultural pesticides has been associated with cochlear oxidative damage and apoptotic cell death [25,26,27,28,29,30,31,32]. Physical factors, including chronic noise exposure and vibration, synergize with chemical pollutants through shared oxidative and inflammatory pathways within the auditory epithelium [15,20,24,33]. Industrial and occupational cohort studies indicate that workers in manufacturing, construction, and transportation sectors have significantly higher odds of sensorineural impairment relative to unexposed counterparts, even after adjusting for age and genetics [34,35,36,37,38]. Collectively, these findings reinforce the multifactorial nature of hearing loss and the need for integrated approaches combining environmental genomics, occupational health surveillance, and targeted preventive interventions.

### 1.2. Multifactorial Nature of Hearing Impairment

The intricate interaction of genetic predisposition, lifelong environmental exposures, and age-related degeneration results in a diverse clinical spectrum of auditory dysfunction [19,22,23,24,29,39]. Twin and family studies consistently estimate that heritable factors account for approximately 50 to 70 percent of variance in hearing thresholds, underscoring the strong genetic input in both age-related and early-onset forms [4,40,41,42]. Genes implicated in cochlear homeostasis, oxidative metabolism, and synaptic maintenance, particularly *GJB2*, *SLC26A4*, *MT-RNR1*, and *OTOF*, are often modulated by concurrent environmental aspects [39,43,44,45,46,47,48]. These genetic factors affect biological pathways that are particularly vulnerable to oxidative stress, mitochondrial dysfunction, and impaired vascularization. This vulnerability highlights the complex interplay between hereditary predisposition and the cumulative effects of environmental and lifestyle-related auditory damage [7,29,49,50,51,52,53,54].

Environmental and occupational factors compound the genetic risk landscape, forming an extrinsic dimension to hearing impairment’s multifactorial nature [7,41,55,56,57]. Prolonged exposure to physical agents, such as high-intensity noise and whole-body vibration, damages sensory hair cells and cochlear neurons through oxidative and excitotoxic mechanisms [33,34,36,38,55,58,59]. Xenobiotics including organic solvents, heavy metals, and ototoxic pharmaceuticals, exert synergistic cytotoxic effects via mitochondrial injury, disruption of calcium homeostasis, and induction of inflammatory cytokines within the cochlea [4,29,59,60,61]. Inflammatory or autoimmune inner-ear pathologies further exacerbate this burden, linking systemic immune dysregulation to auditory tissue degeneration [61,62,63,64,65]. The convergence of these physical, chemical, and biological mechanisms demonstrates that hearing loss is typically not caused by a single factor, but rather is a dynamic condition shaped by the lifelong interaction between genetic and environmental influences [4,6,7,8,28,33,66].

### 1.3. Gene–Environment Interactions in Hearing Loss: Mechanisms of Cochlear Vulnerability and Prevention

Understanding gene–environment interactions is crucial for explaining why individuals show different levels of susceptibility and progression of hearing loss, even when exposed to similar environmental factors [10,17,22,24,41,56,57]. Recent research has shown that the interplay between genetic polymorphisms and environmental stressors (e.g., oxidative damage from noise, heavy metals, ototoxic drugs, or aromatic solvents) determines both the onset and severity of auditory dysfunction [23,29,41,50,55,60,61,67].

Variations in antioxidant and DNA repair genes, including *BRCA1*, *GSTM1*, *CAT*, and *hOGG1*, have been associated with an increased vulnerability to noise-induced and environmentally mediated hearing loss, supporting the biological plausibility that genetic background modulates environmental ototoxicity [68,69,70,71,72,73,74,75,76]. Recognizing these interactions improves the mechanistic understanding of auditory pathology and enables the identification of high-risk/susceptible genotypes in exposed populations, facilitating targeted prevention and more adequate monitoring strategies [22,23,27,28,33,34,35,36,38,40,55,67,77].

Advances in gene-based treatments and epigenetic modulation are under investigation as potential approaches for more precisely directed and better-tailored interventions according to individual genomic profiles, including strategies to restore or preserve cochlear function through adeno-associated virus (AAV)-mediated gene delivery, RNA interference, and antioxidant gene upregulation [67,78,79,80,81,82,83,84]. The integration of genetics, molecular ototoxicology, and occupational health surveillance may represent an important approach for mitigating hearing loss as a global non-communicable disease burden.

Evidence from genetic association and animal studies demonstrates that variations in genes regulating antioxidant capacity, mitochondrial respiration, and potassium ion recycling (among them *SOD2*, *CAT*, *GPX1*, *GSR*, *GST*, and *GJB2*), increase sensitivity to environmental ototoxins [70,71,72,73,74,78,85,86,87,88]. These pollutants generate excessive reactive oxygen species (ROS) within cochlear hair cells and spiral ganglion neurons (SGNs), overwhelming intrinsic scavenging systems and triggering lipid peroxidation, mitochondrial DNA (mtDNA) damage, and apoptosis [29,33,74,87,89,90,91,92]. Individuals harboring genetic variants that impair oxidative defense exhibit markedly greater cochlear vulnerability under identical exposure conditions, reinforcing the concept that genotype determines the threshold for environmentally induced injury [41,55,57,73].

In addition to acting as damaging oxidants, ROS serve as signaling molecules that potentiate inflammatory gene expression through the NF-κB and Nrf2/Keap1 regulatory pathways. Chronic activation of these systems alters cochlear cytokine profiles, with evident upregulation of TNF-α, IL-1β, and IL-6, which promotes macrophage infiltration and, in turn, amplifies cytotoxic stress [61,93,94,95,96]. Models combining cadmium or toluene exposure with partial deletion of *GSTM1* or *HSP70* genes illustrate that oxidative and inflammatory processes are biologically intertwined; the absence of adequate molecular chaperones or detoxifying enzymes precipitates irreversible loss of sensory hair cells and synaptic terminals [69,97,98,99,100,101]. This convergence between redox imbalance and inflammation constitutes a self-perpetuating loop of cochlear degeneration that bridges environmental insult and genetically determined vulnerability [33,55,61,85,88,95].

Beyond ROS and cytokine dysregulation, disruption of mitochondrial bioenergetics emerges as a unifying pathway. Mitochondrial DNA polymorphisms, particularly within *MT-RNR1* and *POLG*, have been linked to both aminoglycoside-induced and pollutant-related ototoxicity, owing to impaired respiratory chain efficiency and decreased ATP availability [45,50,51,52,53,60]. Pollutant-driven oxidative stress further damages mtDNA, leading to defective protein synthesis and activation of the mitochondrial permeability transition pore, culminating in caspase-dependent apoptosis of cochlear cells [29,33,92,102,103]. These findings suggest that mitochondrial genotype not only shapes basal metabolic resilience but also governs how environmental exposures translate into structural auditory injury.

Seminal integrative multi-omics datasets—combining genomics, transcriptomics, and exposomics—are beginning to identify molecular signatures that predict hypersensitivity to pollutants, alterations in oxidative and inflammatory status, and accelerated cochlear degeneration and hearing loss [41,55,57,73,104]. These insights provide a conceptual framework for developing novel interventions, including antioxidant gene therapy and small-molecule modulators targeting mitochondrial stress responses [53,67,105].

## 2. Genetic Basis of Hearing Loss

### 2.1. Genetic Mechanisms Underlying Hearing Loss Susceptibility

**Figure 1:**
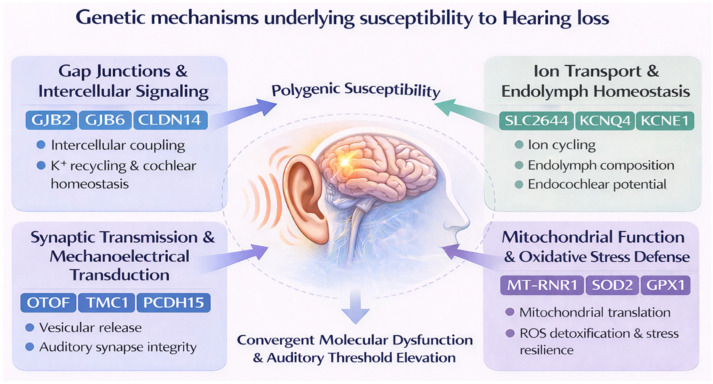
Genetic factors that increase the risk of hearing loss, highlighting clinically and biologically relevant genes involved in intercellular communication, mechanoelectrical transduction, endolymph homeostasis, mitochondrial function and oxidative stress defense that converge to cochlear molecular dysfunction.

The underlying genetic mechanisms of deafness encompass a wide spectrum of molecular pathways that regulate cochlear function, sensory cell survival, and auditory synaptic integrity (Figure 1). More than 150 genes have been implicated in hereditary or environmentally modulated forms of deafness, underscoring the remarkable genetic heterogeneity and biological complexity of auditory physiology [106,107,108,109,110]. A comprehensive and continuously updated list of all known deafness-associated genes, with gene-disease validity classifications assigned using the ClinGen framework, is maintained and available on the Hereditary Hearing Loss Homepage (https://hereditaryhearingloss.org/, accessed on 10 December 2025) [111]. This resource also includes OMIM links, inheritance patterns, *locus* identifiers, and associated syndromes. Given the extensive number of genes involved, only key representative examples of deafness-associated genes are highlighted in Table 1.

Genetic mechanisms of susceptibility extend from congenital mutations causing syndromic and nonsyndromic deafness to subtle sequence variants that predispose individuals to acquired sensorineural decline when exposed to environmental stressors. Common examples include *GJB2*, *SLC26A4*, *OTOF*, and *MT-RNR1*, whose products are essential for intercellular signaling, ion cycling, vesicular neurotransmission, and mitochondrial translation within cochlear hair cells [9,43,44,45,46,48,52,53,78,87,106,146,147,148,149]. Dysfunction in these genes disrupts endolymph homeostasis and impairs mechanoelectrical transduction, ultimately driving auditory threshold elevation and progressive cell death [44,46,47,49,150].

Advances in genome-wide sequencing and multi-omics profiling have expanded the spectrum of candidate genes associated with both congenital and adult-onset hearing loss. Beyond classic Mendelian mutations, genome-wide association studies have identified polygenic contributions involving oxidative stress regulators (*SOD2*, *CAT*, *GPX1*), potassium channel subunits (*KCNQ4*, *KCNE1*), and gap junction proteins (*GJB6*, *CLDN14*), which collectively fine-tune cochlear metabolism and vulnerability to environmental ototoxins [42,85,151,152,153,154,155]. These findings illustrate that even modest genetic perturbations can influence cellular resilience under oxidative or inflammatory conditions, highlighting a transitional continuum between genetic and acquired forms of auditory impairment [55,85,88,156]. While the identification of numerous deafness-associated genes has significantly advanced our understanding of the genetic basis of hearing loss, the sheer number of genes involved poses a challenge for diagnosis and treatment. Further research is needed to elucidate the functional consequences of these genetic variants and their interactions with environmental factors.

### 2.2. Strong Genetic Contribution Modulated by Environmental Exposures

Twin and family studies have consistently demonstrated that age-related hearing loss (ARHL), or presbycusis, has a substantial genetic component, with heritability estimates ranging between 50% and 70% across diverse populations [4,40,42,153,157,158,159,160,161]. Large-scale twin analyses, such as the Vietnam Era Twin Study of Aging, found that genetic factors accounted for approximately 49–68% of the variance in hearing thresholds across frequencies, a proportion that remained stable over more than a decade of longitudinal follow-up [41,162]. The same magnitude was observed in population-based twin studies in China and Sweden, confirming a robust heritable influence particularly at mid- and high-frequency ranges, where hearing loss typically manifests earliest [158,159,160,163,164]. These findings collectively underscore that ARHL is not solely a stochastic byproduct of aging, but a complex phenotype largely shaped by inherited biological predisposition.

Genome- and phenome-wide association studies further suggest that both common and rare variants in genes traditionally linked to monogenic deafness (e.g., *GJB2*, *POU4F3*) can modulate presbycusis risk when acting within polygenic backgrounds. Genetic contributions to ARHL appear to encompass an array of *loci* involved in oxidative stress responses, synaptic transmission, and cochlear homeostasis. Variants in genes such as *GRM7*, *KCNQ4*, *SOD2*, and *GRHL2*, implicated in neurotransmission, ion channel regulation, redox balance, and epithelial integrity, have been associated with gradual threshold shifts across the adult lifespan [41,55,57,73,156,157,165,166,167]. Genome- and phenome-wide association (PheWAS) studies extending these observations revealed that both common and rare variants in genes classically associated with monogenic deafness (e.g., *GJB2*, *POU4F3*) have been explored as potential modifiers of age-related hearing loss risk in population-based studies, although evidence for heterozygous effects remains limited and variant-dependent [42,151,152,153,154,155,157,168,169,170,171].

While genetic factors account for much of baseline auditory function, inter-individual variability in hearing deterioration over time is predominantly shaped by unique environmental exposures (e.g., chronic noise, ototoxic pollutants, smoking, and metabolic disease) [23,24,29,40,41,158,164,167,172]. Longitudinal twin models reveal that nearly 80% of the variance in hearing decline trajectories can be attributed to person-specific environmental influences, even as the genetic baseline remains stable [163]. This dynamic interplay highlights that heritability estimates describe intrinsic vulnerability, but not inevitability. The strong genetic contribution to ARHL highlights the importance of considering genetic factors in the diagnosis and management of hearing loss. However, the remaining variance attributed to environmental influences underscores the need for a comprehensive approach that incorporates both genetic and environmental factors.

### 2.3. Major Genes Associated with Deafness

Mutations in *GJB2*, encoding connexin 26 (Cx26), represent the most frequent congenital cause of autosomal recessive nonsyndromic deafness (DFNB1), accounting for nearly 50% of cases worldwide [18,49,106,113]. Cx26 is a gap junction protein expressed in cochlear supporting cells and is crucial for intercellular signaling, potassium (K^+^) recycling, and metabolic coupling within the organ of Corti [43,173,174,175]. Stablished *GJB2* pathogenic variants account for 18–50% of prelingual nonsyndromic hearing loss globally, with the c.35delG variant alone responsible for approximately 60–70% of deafness in many populations, including European, North African, Middle Eastern, and American cohorts [47,49,113,176,177]. Loss-of-function mutations, such as 35delG or 167delT, disrupt gap junction permeability, impairing ionic and nutrient transport necessary for maintaining the endocochlear potential [173,174,178,179]. This leads to reduced outer hair cell electromotility, compromised cochlear amplification, and ultimately sensorineural dysfunction [29,33,91,180]. Moreover, connexin deficiency reduces glucose diffusion across the avascular sensory epithelium, resulting in mitochondrial energy deficits and accumulation of ROS [173,178,181].

The solute carrier family 26, member 4 gene (*SLC26A4*), which encodes pendrin, is responsible for the second most common form of autosomal recessive hereditary hearing loss and is associated with Pendred syndrome and enlarged vestibular aqueduct (EVA) [115,147,149,182,183]. Pendrin is an anion exchanger localized to the apical membrane of epithelial cells in the endolymphatic sac and cochlear duct, where it mediates chloride/bicarbonate exchange, critical for maintaining ionic composition and endolymph pH. Mutations impair this delicate ionic balance, leading to osmotic stress, endolymphatic hydrops, and degeneration of hair cells secondary to inflammatory and oxidative signaling [184,185,186,187,188]. Experimental models demonstrate that *Slc26a4*-null mice exhibit aberrant strial morphology, oxidative stress activation, and loss of endocochlear potential before hair-cell apoptosis [189,190]. Moreover, low-grade inflammation exacerbates pendrin-related pathology, supporting the emerging concept that chronic immune microactivation in the inner ear amplifies genetic susceptibility to environmental or metabolic insults [185,191,192], a topic developed further in Section 4.

Mutations in *OTOF*, encoding otoferlin, disrupt synaptic transmission between inner hair cells (IHCs) and auditory nerve fibers. Otoferlin acts as a calcium sensor essential for synaptic vesicle fusion with the presynaptic plasma membrane, facilitating rapid neurotransmitter release at the ribbon synapse [193,194]. Loss-of-function mutations cause auditory neuropathy spectrum disorder (ANSD or DFNB9), characterized by preserved outer hair cell function but defective sound transduction downstream of the IHCs [9,46]. Patients harboring specific *OTOF* variants typically present early-onset, stable, and moderate-to-severe or profound deafness; however, certain alleles increase vulnerability to environmental challenges, such as febrile episodes (e.g., c.1544T > C (p.Ile515Thr) or c.2485C > T (p.Arg829Trp)) or putatively linked to acoustic stress (e.g., c.4024G > A (p.Glu1342Lys) or c.5473C > T (p.Arg1825Trp)) [118,195,196,197,198]. Experimental evidence also suggests that otoferlin deficiency heightens synaptic oxidative stress and calcium dyshomeostasis during noise exposure, aggravating cochlear excitotoxicity [199,200,201]. Gene supplementation using adeno-associated viral vectors/AAV systems has recently achieved partial rescue of auditory responses in *Otof*-deficient mice, illustrating the translational relevance of this mechanism [202]. Thus, emerging evidence on the role of otoferlin in synaptic transmission and the potential for gene therapy to restore auditory function is promising (human *OTOF* AAV therapy is discussed further in Section 5.2. Gene therapy and epigenetic approaches).

Lastly, mitochondrial variants such as those within mitochondrially encoded 12S rRNA (*MT-RNR1*), mitochondrially encoded tRNA serine 1 (*UCN* or *MT-TS1*), and DNA polymerase gamma, catalytic subunit (*POLG*), are key modifiers of susceptibility to pharmacological and environmental ototoxicity [45,203,204,205]. The well-established *MT-RNR1* m.1555A > G and m.1494C > T mutations predispose individuals to irreversible aminoglycoside-induced hearing loss, owing to increased antibiotic-ribosome binding affinity and mitochondrial protein synthesis failure [45,50,51]. These alterations impair oxidative phosphorylation, elevate ROS generation, and compromise energy supply to cochlear hair cells, which are particularly dependent on mitochondrial metabolism [51,52,53,60,102,206,207,208,209,210,211]. Further research is needed to clarify how major deafness-associated genetic variants, including mitochondrial variants linked to ototoxicity, influence hearing loss and to inform effective therapeutic strategies. A nuanced understanding of the complex interplay between genetic and environmental factors is essential for improving the diagnosis and management of hearing loss.

### 2.4. Genes Related to Antioxidant Defense

Variants in genes encoding antioxidant defense enzymes, including superoxide dismutase 2 (*SOD2*), catalase (*CAT*), glutathione peroxidase 1 (*GPX1*), and NAD(P)H quinone oxidoreductase 1 (*NQO1*), have emerged as key determinants of cochlear vulnerability to oxidative injury [87,212,213,214,215]. These enzymes constitute the core of the endogenous defense system that neutralizes ROS generated during mitochondrial respiration, inflammation, and noise exposure [29,33,90,91,216]. SOD2 enzyme, localized in mitochondria, catalyzes the dismutation of superoxide radicals into hydrogen peroxide, which is subsequently decomposed by catalase and glutathione peroxidases. Deficiency or genetic polymorphism in *SOD2*, notably the Val16Ala variant, has been associated with reduced enzymatic efficiency, enhanced ROS accumulation, and heightened susceptibility to both age-related and noise-induced hearing loss [89,217,218,219,220,221]. Similarly, *CAT* polymorphisms alter the conversion of hydrogen peroxide to water and oxygen, leading to oxidative damage in cochlear hair cells under acoustic and ototoxic stress [29,33,87,90,91]. Deficient catalase activity amplifies mitochondrial dysfunction and lipid peroxidation within outer hair cells (OHCs), promoting apoptotic pathways that culminate in progressive auditory decline [61,70,73,93,94,95,96].

The GPX1 and NQO1 enzymes play complementary roles in maintaining redox equilibrium and controlling inflammatory signaling. Variants such as *GPX1 Pro198Leu* are linked to decreased detoxification of hydrogen peroxide and organic hydroperoxides, amplifying cochlear oxidative stress and vulnerability to ototoxins [41,55,57,214,222]. NQO1, a multifunctional flavoprotein enzyme regulated by the Nrf2-ARE pathway, catalyzes the two-electron reduction of quinones to hydroquinones, thereby limiting redox cycling and contributing to intracellular redox balance, including NAD(P)H homeostasis [223,224]. Experimental induction of *NQO1* expression has been shown to attenuate age-related auditory threshold shifts in rodent models by sustaining mitochondrial biogenesis and restraining NF-κB- and p53-mediated inflammatory cascades [215,223,225]. Collectively, these findings demonstrate that minor polymorphisms in genes encoding antioxidant defense mechanisms, such as *GPX1* c.593C > T (p.Pro198Leu, rs1050450) and *NQO1* c.559C > T (p.Pro187Ser, rs1800566), can significantly modulate individual sensitivity to environmental ROS, supporting a role for redox gene variants as important modulators of gene–environment interactions in hearing loss [87,90,91,95,96,216].

The role of antioxidant defense genes in modulating susceptibility to hearing loss highlights the importance of considering the interplay between genetic and environmental factors. Genetic variation in these pathways influences the capacity to counteract oxidative stress, thereby shaping individual risk profiles for cochlear damage and hearing impairment.

### 2.5. Additional Deafness-Associated Genes

Beyond the classical deafness genes (e.g., *GJB2*, *SLC26A4*, *OTOF*, *MT-RNR1*) and antioxidant *loci* (e.g., *SOD2*, *CAT*, *GPX1*, *NQO1*), an additional set of genes has been recognized as central to the genetic heterogeneity of sensorineural hearing loss. Among these, structural and cytoskeletal genes such as *STRC* and *TECTA* play pivotal roles in maintaining stereocilia organization and tectorial membrane integrity. *STRC* deletions causing DFNB16, constitute up to 15% of mild-to-moderate autosomal recessive deafness cases worldwide [106,130,131]. Similarly, *TECTA* and *COL11A2* mutations result in autosomal dominant forms of mid-frequency or progressive hearing loss due to altered extracellular matrix composition in the cochlear tectorial membrane [126,134,226,227].

Other *loci*, including actin-motor and synaptic genes such as *MYO6*, *POU4F3*, *EYA4*, and *TBC1D24*—mediate cochlear hair cell mechano-transduction and vesicular trafficking —and specific mutations in *MYO6* and *POU4F3* are consistently associated with DFNA autosomal-dominant progressive hearing loss and age-dependent neuronal dysfunction [106,134,228,229]. Furthermore, *COCH* and *WFS1* defects illustrate genotype–phenotype intersections between auditory and vestibular impairment, the former manifesting as late-onset progressive hearing loss accompanied by episodic vertigo and tinnitus [142,229,230,231]. X linked deafness, though rare, is exemplified by *POU3F4* mutations (DFNX2), resulting in stapes fixation and perilymphatic gusher during cochlear implantation [232,233].

Collectively, these findings underscore that nonsyndromic hearing loss is profoundly polygenic, with >100 confirmed causative genes spanning pathways of ionic homeostasis, cytoskeletal assembly, and synaptic maintenance [42,151,152,153,154,155,163,170]. The elucidation of these networks not only improves molecular diagnosis but also broadens the pathophysiological continuum between hereditary and environmentally modulated auditory dysfunction. The identification of novel deafness-associated genes continues to expand our understanding of the complex genetic landscape of hearing loss. The functional characterization of these genes and their interactions with environmental factors remains a significant challenge.

### 2.6. GWAS Major Achievements and Breakthroughs

Genome-wide association studies (GWAS) have revolutionized the understanding of polygenic contributors to hearing loss susceptibility, uncovering a spectrum of common and rare variants that collectively shape individual vulnerability [134,154,171,234]. Early GWAS in large biobanks such as the UK Biobank and deCODE Icelandic cohort identified over 50 independent genomic *loci* linked to age-related hearing impairment (ARHI), many within or proximal to known Mendelian deafness genes, including *KCNQ4*, *POU4F3*, *GRM7*, and *OTOF* [42,151,152,153,155,235].

Pathway analyses connected these variants with mechanosensory transduction, synaptic signaling, and actin-cytoskeletal regulation, confirming biological overlap between monogenic and complex auditory phenotypes [151,235]. A meta-analysis encompassing >720,000 participants across 17 independent cohorts expanded this number to 48 significant *loci*, including 10 novel associations, implicating genes expressed in SGNs and the stria vascularis, structures essential for endocochlear potential maintenance [154]. More recently, the 2025 Million Veteran Program (MVP) and UK Biobank meta-GWAS identified 108 *loci* and 54 novel genes, representing the most comprehensive genetic architecture of adult sensorineural hearing loss to date [236]. Particularly, while 97% of risk variants occurred outside classical hereditary hearing loss (HHL) genes, their expression was enriched in cochlear hair cells, neurons, and stereocilia rootlets, underscoring that multigenic susceptibility converges on shared mechano-transductive and mitochondrial processes.

Collectively, these milestones have enabled the development of genetic risk scores (GRS) capable of stratifying populations by lifetime risk of auditory disease, establishing GWAS as a cornerstone in connecting genomic discoveries to precision otology [134,154,234,237]. Nevertheless, while GWAS have identified numerous genetic variants associated with hearing loss, the translation of these findings into clinically relevant insights remains an ongoing challenge. Further research is needed to elucidate the functional consequences of these variants and their potential as therapeutic targets.

### 2.7. Epigenetic Regulation

Epigenetic regulation has emerged as a crucial layer influencing the expression of auditory genes and susceptibility to environmental insults without altering the primary DNA sequence. DNA methylation of promoter regions in antioxidant and stress-response genes, including *SOD2*, *CAT*, and *GPX1*, modulates transcriptional activity in response to oxidative or acoustic stress [159,238,239,240,241]. Aberrant promoter hypermethylation diminishes gene transcription, reducing antioxidant enzyme capacity and leading to increased cochlear oxidative burden and apoptotic signaling [242,243,244]. For instance, exposure to chronic noise or ototoxic compounds has been shown to induce hypermethylation in cochlear tissues and peripheral blood DNA, in parallel with down-regulation of endogenous antioxidant pathways [238,245]. Conversely, pharmacological interventions using DNA-methyltransferase (DNMT) inhibitors and histone deacetylase (HDAC) blockers can partially restore the transcriptional activity of silenced protective genes, highlighting both the reversibility of these epigenetic modifications and their significance for prevention strategies and targeted treatment approaches [238,244,246].

MicroRNAs (miRNAs) constitute another dimension of epigenetic regulation that fine-tunes gene expression post-transcriptionally and dynamically responds to environmental stress [247,248]. miRNAs, such as miR-34a or miR-29b, are upregulated in cochlear tissues following oxidative or inflammatory stimuli, where they target antioxidant-related and pro-survival genes [249,250,251]. The *miR-34a*, a p53-responsive miRNA, inhibits *SIRT1*, a critical regulator of mitochondrial biogenesis and ROS detoxification, thereby accelerating hair-cell senescence and apoptosis in response to noise or drug injury [250,252]. Similarly, *miR-29b* suppresses extracellular matrix and mitochondrial genes involved in redox balance, linking its overexpression with elevated cochlear ROS and inflammation during acoustic stress [249,251,253]. The reduction in these deleterious miRNAs, through antagomir approaches or epigenetic modulators such as HDAC inhibitors, rescues auditory thresholds and preserves hair-cell morphology in experimental models [83,244,246]. These insights emphasize that epigenetic events, particularly promoter methylation and miRNA dysregulation, seem to shape cochlear gene expression landscapes, oxidative homeostasis, and ultimately auditory longevity [238,246,247,248].

The study of epigenetic mechanisms in hearing loss is a rapidly evolving field that holds promise for the development of novel therapeutic strategies. However, the complex interplay between epigenetic regulation, genetic factors and environmental influences in hearing loss remains poorly understood.

## 3. Environmental Determinants of Hearing Loss

### 3.1. Ototoxic Agents and Environmental Xenobiotics

Environmental contributions to auditory dysfunction extend beyond acoustic overexposure and encompass a substantial range of xenobiotic pollutants with well-characterized ototoxic potential (Table 2). Organic solvents such as benzene, toluene, xylene, and styrene are well-established ototoxicants capable of disrupting both cochlear and neural components of the auditory system. These lipophilic compounds easily, penetrate biological membranes and cross the blood–labyrinth barrier, accumulating within the stria vascularis and the OHCs, where they interfere with ionic regulation and metabolic homeostasis [24,28,29,32,59,254]. With some frequency, their metabolism generates ROS through cytochrome P450 (CYP)-mediated oxidative biotransformation, leading to lipid peroxidation, membrane disintegration, and apoptosis of cochlear hair cells [28,32,255]. Experimental models reveal significant loss of OHCs and degeneration of SGNs following combined solvent and noise exposure, confirming that chemical ototoxicity often interacts synergistically with acoustic stress [24,29,93,99]. Epidemiological studies among industrial and aviation workers have demonstrated increased odds of high-frequency hearing loss in individuals with elevated blood biomarkers of benzene, ethylbenzene, and toluene after adjustment for covariates such as age and occupational noise [33,256,257,258,259,260]. Collectively, these studies reveal that solvent ototoxicity involves multi-site injury to hair cells, the stria vascularis, and the auditory cortex, mediated primarily by oxidative stress and mitochondrial dysfunction.

Heavy metals such as lead, mercury, and cadmium represent another major class of environmental ototoxins that compromise cochlear and neural physiology through sustained oxidative and inflammatory processes. These metals accumulate in the inner ear’s soft tissues, alter calcium homeostasis, and interfere with mitochondrial electron transport, resulting in excessive ROS production and ATP depletion [7,24,27,60,95]. Chronic low-level exposure has been correlated with disrupted antioxidant enzyme activity, particularly glutathione peroxidase and catalase, thereby amplifying oxidative injury and apoptosis in cochlear hair cells [29,33,70,90,91,261]. Pesticides and organophosphates induce pro-inflammatory cascades via NF-κB activation and excessive nitric oxide formation, leading to degeneration of auditory synapses and SGNs. These compounds may potentiate ototoxic and neurotoxic outcomes when combined with limited antioxidant defenses or concurrent noise exposure [262,263,264,265].

**Table 2:** Environmental pollutant and pharmaceutical xenobiotics associated with hearing loss. Xenobiotic class, evidence, cellular and molecular mechanisms and targets.

Xenobiotic Class [Examples]	Evidence (Typical Patterns/Interactions)	Main Cellular & Molecular Mechanisms	Key Cochlear/Neural Targets	Relevant References
**Aromatic & industrial organic solvents (VOCs)** **[BTEX mixtures; Benzene, toluene, ethylbenzene, xylene]**	Occupational and population studies associate higher biomarker levels with hearing disorders; synergy with noise is common	Lipophilic uptake and blood–labyrinth barrier passage; accumulation in stria vascularis and OHCs; CYP oxidative biotransformation → ROS, lipid peroxidation; mitochondrial dysfunction, ATP depletion; apoptosis	OHCs; stria vascularis; auditory nerve/central pathways	[28,29,32,254,256,258,260,266]
**Industrial organic solvents** **[Styrenics–styrene]**	Animal/experimental evidence of cochlear injury; epidemiology supports increased risk, especially with co-exposure to noise	ROS generation (incl. metabolic activation); oxidative stress and mitochondrial injury; membrane damage; hair cell apoptosis; potentiation of acoustic trauma	OHCs; SGNs	[28,256,257,258,259]
**Mixtures: solvents + noise** **[TEXS mixtures; toluene/ethylbenzene/xylene/styrene]**	Co-exposure studies show greater threshold shifts than single exposures; consistent “adding insult to injury” pattern	Convergent oxidative stress pathways; exacerbated mitochondrial dysfunction; greater synaptopathy/SGN vulnerability under metabolic stress	OHCs; ribbon synapses; SGNs	[256,266,267,268]
**Heavy metals** **[Lead (Pb)]**	Meta-analytic and cohort evidence links blood Pb to hearing loss; experimental work shows synaptic injury and potentiation of NIHL	Accumulation in inner ear tissues; Ca^2+^ dysregulation; mitochondrial electron transport interference → ROS/ATP depletion; antioxidant enzyme disruption (e.g., GPx, catalase); nitrative stress; inflammation; apoptosis	Ribbon synapses; OHCs; stria; SGNs	[7,24,26,27,261]
**Heavy metals** **[Mercury (Hg)]**	Epidemiology supports association with auditory dysfunction (strength depends on exposure metrics and co-exposures)	Oxidative stress; mitochondrial dysfunction; Ca^2+^ homeostasis disruption; inflammatory signaling	Hair cells; SGNs	[24,25,27]
**Heavy metals** **[Cadmium (Cd)]**	Epidemiologic associations reported with auditory impairment; often alongside smoking/occupational co-exposures	Oxidative stress; mitochondrial impairment; depletion of antioxidant defenses; inflammatory mediators	Hair cells; stria vascularis	[24,25,27]
**Pesticides: organophosphates (OPs)** **[chlorpyrifos and other OP insecticides]**	Human studies in agricultural settings show elevated high-frequency thresholds (meaning hearing worsens); risk increases with noise and other exposures	NF-κB activation, pro-inflammatory cascades; excess NO/iNOS signaling; oxidative/nitrative stress; synaptic degeneration; neurotoxicity to SGNs	Ribbon synapses; SGNs; possible efferent pathway disruption	[38,262,263,264]
**Pesticides: organochlorines (persistent organic pollutants-POPs)** **[legacy POPs measured in serum]**	NHANES-based association reported between organochlorine body burden and hearing impairment	Likely oxidative stress, endocrine/mitochondrial disruption and neuroinflammation (mechanistic evidence is less direct than for solvents/metals)	Cochlea + auditory neural pathways	[24,25,265]
**Combined chemical mixtures in workplaces** **[Metals + solvents + noise (various)]**	Studies in shipyards/industry indicate higher risk and permanent threshold shifts (hearing loss) under multi-exposure scenarios	Additive/synergistic oxidative stress and inflammatory signaling; reduced antioxidant reserve; compounded metabolic load	OHCs; stria; SGNs	[24,267,269]
**Pharmaceutical xenobiotics: ribosome-targeting ototoxic antibiotics** **[aminoglycosides (e.g., gentamicin; class-level)—exposure in carriers of MT-RNR1 m.1555A > G/m.1494C > T]**	Irreversible progressive SNHL can occur after transient aminoglycoside exposure, including at apparent “clinically safe” doses in MT-RNR1 carriers; vulnerability can be amplified by co-stressors (e.g, noise/acoustic trauma, smoking, metabolic syndrome)	Enhanced aminoglycoside binding to mitochondrial 12S rRNA (MT-RNR1 variants) → impaired mitochondrial protein synthesis and oxidative phosphorylation; ROS overproduction; altered NAD^+^/NADH balance; Ca^2+^ overload; intrinsic apoptosis (cytochrome *c* release, caspase-9 activation); mtDNA damage	Cochlear hair cells (mitochondria-dependent, especially OHCs); downstream synapses/neurons	[45,50,51,52,53,60,89,93,102,206,207,208,209,210,211,270,271,272]
**Pharmaceutical xenobiotics: other ribosome-targeting ototoxic antibiotics** **[non-aminoglycosides (e.g., macrolides as erythromycin)]**	SNHL has been reported after exposure to non-aminoglycoside antibiotics and may be modulated by co-stressors (e.g, noise/acoustic trauma, smoking, metabolic syndrome) and host susceptibility (mtDNA variants, including carriers of MT-RNR1 m.1555A > G/m.1494C > T)	Conserved antibiotic–ribosome interaction motifs suggest possible off-target disruption of mitochondrial ribosomes/translation; reduced mitochondrial protein synthesis impairs oxidative phosphorylation and ATP supply, elevates ROS and redox imbalance, perturbs Ca^2+^ homeostasis, and activates intrinsic apoptosis (cytochrome c release → caspase-9), with mtDNA damage and defective mitochondrial maintenance further amplifying injury	Mitochondria-dependent cochlear hair cells (esp. OHCs); stria vascularis; downstream synapses/SGNs (secondary vulnerability)	[89,92,102,206,207,208,209,210,211,222,270,272,273,274]

NHANES: National Health and Nutrition Examination Survey (U.S.).

The evidence linking ototoxic agents and environmental pollutants to hearing loss is substantial, but the complexity of real-world exposures, involving mixtures of xenobiotics and interactions with other factors such as noise, complicates the establishment of clear causality and dose–response relationships. Further work is needed to elucidate the mechanisms underlying these interactions and to inform evidence-based prevention and mitigation strategies.

### 3.2. Noise Exposure and Occupational Ototoxicity

Occupational and environmental noise exposure remains one of the most pervasive risk factors for sensorineural hearing loss worldwide, affecting millions of workers annually. Prolonged exposure to intense acoustic stimuli, typically exceeding 75 dBA, refs. [1,275,276] over time increases the risk of noise-induced hearing loss (NIHL), a preventable but common occupational illness. This exposure induces metabolic exhaustion, oxidative stress, and inflammatory cascades within cochlear tissues [22,30,33,36,37]. Excessive sound energy leads to mechanical disruption of stereocilia and persistent generation of reactive oxygen and nitrogen species (RNS), which damage mitochondrial membranes and activate apoptotic pathways in hair cells and SGNs [277,278,279,280]. As oxidative stress progresses, inflammatory transcription factors such as NF-κB seem to be upregulated, driving production of cytokines, which includes TNF-α and IL-1β that further compromise cochlear blood flow and cellular integrity [281,282]. Persistent inflammation and redox imbalance establish a self-sustaining cycle of cellular degeneration that contributes to permanent threshold shifts observed in occupational and environmental NIHL [33,282].

Combined exposures to noise and ototoxic chemicals, including organic solvents and heavy metals, exacerbate auditory damage through convergent activation of oxidative and pro-inflammatory molecular pathways. Experimental and epidemiological studies demonstrate that such co-exposures amplify lipid peroxidation and mitochondrial injury relative to either agent alone, underscoring a synergistic model of cochlear toxicity [29,267,269,283]. Central to this synergism is the crosslink between the Nrf2/Keap1 antioxidant defense pathway and NF-κB-driven inflammatory signaling. While Nrf2 activation normally promotes detoxification and redox balance, chemical stressors and continuous acoustic overstimulation attenuate its transcriptional responsiveness, allowing unchecked ROS accumulation and cytoskeletal degradation [83,95,96,284]. This mechanistic interplay provides the molecular basis for the heightened cochlear susceptibility observed among industrial workers simultaneously exposed to solvents, heavy metals, and/or noise [22,23,29,35,267,283]. Therefore, recognition of combined ototoxic hazards emphasizes the importance of integrated workplace strategies that extend beyond decibel reduction to include chemical risk management and antioxidant-based preventive interventions. While noise exposure is a well-established risk factor for hearing loss, the variability in individual susceptibility and the impact of co-exposures to other ototoxic agents highlight the need for a more nuanced understanding of the complex interactions underlying NIHL.

### 3.3. Experimental and Epidemiological Evidence

Experimental and epidemiological research collectively provide evidence that combined exposure to noise and solvents accelerates auditory damage more severely than either agent alone. Early studies using rodent model demonstrated that simultaneous exposure to aromatic solvents such as toluene, styrene, benzene, or trichloroethylene and moderate acoustic overexposure caused greater auditory threshold shifts, cochlear lipid peroxidation, and hair cell apoptosis, than single exposures [29,256,257,266,268]. Morphological analyses revealed degeneration of OHCs and SGNs, along with increased mitochondrial swelling and degeneration in the organ of Corti [93,99,103,279]. These findings paralleled mechanistic insights showing an overload of ROS and disruption of calcium homeostasis, which can be attributed to the combined activation of oxidative and inflammatory cascades [33,90,91]. The synergistic ototoxic effects were observed even when noise levels were below safety thresholds (75dBA), supporting the hypothesis that solvent exposure sensitizes the cochlea to sound injury through oxidative mechanisms and Nrf2/Keap1 pathway suppression. These experimental data underscore a dose-dependent and synergistic interaction between noise energy and solvent exposure, leading to accelerated apoptotic cell death in cochlear tissues [29,95,257].

Human evidence from industrial and occupational cohort studies aligns closely with these laboratory findings, confirming the synergistic auditory hazard of combined exposures. Epidemiological analyses across manufacturing, aviation, and printing industries consistently report higher hearing-loss prevalence among workers co-exposed to noise and solvents, with adjusted odds ratios typically ranging from 2.1 to 3.0 compared with noise-only exposure [23,28,29,32,38,256,285]. A large meta-analysis of thirteen human studies found that 43% of participants exposed to both solvents and noise exhibited measurable auditory pathology, compared with 24.5% in noise-only groups, yielding a pooled odds ratio of 2.75 (*p* < 0.001) [256]. Recent cohort investigations in shipyard and petrochemical workers revealed that long-term mixed exposure significantly worsened high-frequency hearing thresholds and increased audiometric notch occurrence, even after controlling for age and tenure [256,266,269,285]. While solvent-only exposure produces mild or reversible effects, co-exposure with occupational noise results in persistent sensorineural deterioration and higher rates of irreversible hair-cell apoptosis, reflecting additive oxidative and neurotoxic stress [29,99,256,257,268]. Consequently, both animal and human data converge toward a unified model of synergistic cochlear toxicity, validating combined exposure as a critical determinant of occupational noise-hearing impairment.

The convergence of experimental and epidemiological evidence supporting the synergistic effects of combined noise and xenobiotic exposure on hearing loss highlights the need for a more comprehensive approach to occupational health and safety. Regulatory frameworks and workplace practices should be re-evaluated to account for the potential interactions between noise and ototoxic chemicals and to develop effective strategies for mitigating these risks.

## 4. Physiopathological Mechanisms and Cochlear Damage

### 4.1. Oxidative Stress

ROS represent a central pathogenic mediator in cochlear damage, acting as both initiators and amplifiers of oxidative and inflammatory cascades. In the inner ear, excessive ROS production, typically derived from imbalanced mitochondrial respiration, NADPH oxidase activation, and inflammatory signaling, may result in extensive damage to membrane phospholipids, mtDNA, and structural proteins critical for inner and outer hair cell viability [33,60,61,89]. Following intense noise exposure or ototoxic insult, free radical accumulation occurs within minutes in the spiral ligament and organ of Corti, preceding morphological evidence of cellular death [286,287]. This immediate oxidative state, normally characterized by elevated superoxide, hydroxyl radical, and peroxynitrite production, targets mitochondrial membranes, leading to lipid peroxidation and disruption of the endocochlear potential [29,59,93,242]. Persistent imbalanced levels of ROS impair DNA repair processes and deplete antioxidant defenses, and this may lead to the conversion of initial reversible oxidative modifications into irreversible apoptotic injury, particularly in the base of the cochlea a high-frequency region, notorious for their limited vascularization and antioxidant capacity [33,40,249].

Within cochlear cells, mitochondrial dysfunction is a pivotal consequence of oxidative stress and the main driver of cell death cascades. Excessive levels of ROS seem to cause inhibition of the mitochondrial respiratory chain, particularly complexes I and III, resulting in diminished ATP synthesis and further superoxide dispersion [206,270,288]. These redox-induced impairments initiate mitochondrial depolarization and the opening of the mitochondrial permeability transition pore (mPTP), which facilitates cytochrome *c* leakage into the cytoplasm [89,93]. Released cytochrome *c* interacts with Apaf-1 and procaspase-9 to form apoptosomes, thereby activating the caspase-dependent apoptotic cascade responsible for hair cell and spiral ganglion neuron apoptosis [271,273,289,290]. Moreover, recent transcriptomic and histological studies have confirmed that oxidative stress also triggers regulated necrosis pathways in the cochlea, namely ferroptosis and necroptosis, further amplifying tissue degeneration [93]. This mechanistic complexity reinforces that mitochondrial failure is not merely a downstream endpoint but a dynamic regulatory node in oxidative cochlear injury.

The cascade of mitochondrial disruption and programmed cell death forms the molecular foundation for progressive, irreversible sensorineural hearing loss. Studies in murine models showed that administration of antioxidants such as N-acetylcysteine or coenzyme Q10 (CoQ10) restored mitochondrial membrane potential and reduced both mtDNA oxidation and hair cell loss, apparently confirming that ROS play a direct role causing the injury not causing a side effect [288,291,292]. Complementary clinical and laboratory data indicate that oxidative injury in the organ of Corti coexists with activation of redox-sensitive transcription factors and its dual effect, e.g., NF-κB and Nrf2, which initially promotes protective gene expression but becomes dysregulated during sustained oxidative stress [93,94,95,225]. This dysregulation perpetuates metabolic exhaustion, chronic inflammation, and apoptotic turnover of sensory epithelium, effectively linking transient ROS overproduction to lasting auditory dysfunction. Taken together, findings across animal models and human clinical studies position oxidative stress at the center of cochlear pathophysiology, whereby mitochondrial breakdown and caspase activation represent the final executors of auditory cell death.

### 4.2. Mitochondrial Pathways

Ototoxic xenobiotics, particularly antibiotics, can precipitate hearing loss in a genotype-dependent manner, with mitochondrial variants modulating cochlear bioenergetics and redox homeostasis (Table 2). Carriers of specific mtDNA variants exhibit significantly heightened vulnerability to environmental ototoxic factors due to mitochondrial dysfunction’s central role in cochlear energy metabolism and redox equilibrium. Among these, the *MT-RNR1* m. 1555A > G mutation is the most emblematic, as previously described, conferring extreme susceptibility to aminoglycoside-induced hearing loss even at clinically safe doses [45,50,51,52]. This variant enhances the binding affinity between mitochondrial 12S rRNA and aminoglycoside molecules, thereby disrupting mitochondrial ribosomal fidelity, impairing oxidative phosphorylation, and promoting the overproduction of ROS in hair cells [52,53,93,274]. Carriers of *MT-RNR1* mutations experience irreversible sensorineural hearing loss following transient antibiotic exposure, a pathophysiological response amplified when environmental stressors such as acoustic trauma, smoking, or metabolic syndrome are present [45,51,53]. At the cellular level, aminoglycosides can rapidly compromise mitochondrial metabolism in high-frequency cochlear outer hair cells, further supporting a primary vulnerability of energy-demanding sensory epithelia to antibiotic-triggered bioenergetic collapse and ROS accumulation [284]. Similarly, mutations in the chromosomal gene *POLG* affecting mitochondrial DNA polymerase γ activity compromise mtDNA replication fidelity and repair, leading to progressive accumulation of mtDNA deletions and oxidative lesions that overlap mechanistically with aging- and pollution-related cochlear degeneration [29,102,204].

Functional studies in murine and human models demonstrate that such mitochondrial defects produce accelerated, irreversible auditory decline when combined with environmental ototoxins. In *PolgA*-mutant mice, chronic low-level ROS exposure or moderate noise induces early synaptic loss, increased lipid peroxidation, and rapid hair-cell apoptosis compared with wild-type models [293,294,295]. Parallel findings in patients with *MT-RNR1* m.1555A > G and m.3243A > G mutations indicate that progressive hearing threshold elevation may occur independently of age of onset. These findings support the hypothesis that mitochondrial heteroplasmy, in conjunction with cumulative oxidative load, plays a critical role in determining disease severity [45,51,52,102,205,296]. These variants lead to a redox dysregulation in the cochlea via altered NAD^+^/NADH balance, calcium overload, and activation of intrinsic apoptosis pathways mediated by cytochrome *c* release and caspase 9 activation, as previously described [89,272]. Mechanistically, convergence on mitochondrial permeability transition and intrinsic cell-death signaling provides a plausible common endpoint through which diverse ototoxic stressors—including xenobiotics that interfere with translation and oxidative phosphorylation—can trigger irreversible hair-cell loss [92,288]. Compounding this genetic susceptibility, deficiencies in antioxidant defenses, including variants in *SOD2*, *GPX1*, and *NQO1*, further predispose affected individuals to severe cochlear injury upon pollutant or drug exposure [50,51,71,74,87,88,90]. Collectively, these synergistic genetic–xenobiotic/environmental interactions suggest that mitochondrial genotypes are important modifiers of auditory resilience, which may help explain the variability in hearing-loss progression among individuals exposed to similar environmental conditions. Importantly, antibiotic-associated ototoxicity may extend beyond aminoglycosides through convergent mitochondrial and oxidative stress pathways that compromise cochlear bioenergetics, redox homeostasis, and intrinsic apoptosis signaling, thereby increasing vulnerability of metabolically demanding sensory epithelia (Table 2) [206,207,208,209,210,211,222,274].

### 4.3. Secondary Inflammation

ROS not only act as direct inducers of oxidative damage but also function as pivotal signaling molecules that perpetuate secondary inflammation within the cochlea [61,93,96]. Following acoustic or chemical insult, ROS activate redox-sensitive transcription factors, particularly NF-κB, through upstream mediators such as toll-like receptor 4 (TLR4) and Myeloid differentiation primary response 88 (MyD88), thereby initiating pro-inflammatory transcriptional programs that amplify cochlear injury [94,281,297,298]. In sensory and supporting cells, activated NF-κB induce the expression of cytokines, including TNF-α, IL-1β, and IL-6, along with adhesion molecules that promote macrophage infiltration into the spiral ligament and sensory epithelium [61,93,96,299,300]. This sustained paracrine inflammatory signaling exacerbates oxidative stress and consequently triggers endothelial dysfunction and microvascular constriction, which together impair metabolic recovery and inhibit cochlear self-repair mechanisms [90,192].

Studies using animal models have demonstrated upregulation of TLR-4, TNF-α, and IL-6 within hours of noise exposure, accompanied by recruitment of pro-inflammatory macrophages and prolonged sensory cell degeneration despite cessation of the initiating stressor [280,281,299,301,302]. These findings define a vicious molecular loop in which ROS-driven NF-κB activation sustains cytokine release, macrophage recruitment, and progressive cochlear damage, ultimately preventing functional recovery of the auditory epithelium.

## 5. Translational and Integrated Therapeutic Perspectives

### 5.1. Precise Diagnosis and Risk Prediction

The advent of comprehensive genetic screening panels has significantly advanced precision medicine in hearing loss by enabling early identification of susceptibility variants that predispose individuals to environmentally induced or exacerbated cochlear damage [109,303,304]. Contemporary next-generation sequencing (NGS) approaches, including targeted multigene panels, whole-exome sequencing (WES), and whole-genome sequencing (WGS), enable routine evaluation of more than 150 to 200 genes implicated in syndromic and nonsyndromic forms of hearing loss [109,110,120,123,128,305,306].

Recent large-scale targeted sequencing efforts—e.g., a 227-gene panel in a Chinese cohort (diagnostic yield 57.3%), a WES-based panels of 120–230 genes in European and pediatric cohorts (diagnostic yield 44–50%), a comprehensive gene panel testing in children with an overall diagnostic yield of 44% and causative variants involving 41 genes, and a genetic diagnosis study of sensorineural hearing loss in adults using 196 genes (OTOgenics v3) or 229 genes (OTOgenics v4) related to syndromic and non-syndromic hearing loss (yields ~23% solved cases)—confirm that modern NGS allows routine evaluation of hundreds of hearing-loss genes, substantially increasing diagnostic yield compared to single-gene testing [307,308,309,310]. Thus, panels such as OtoSeq^®^, OtoSCOPE, and the Comprehensive Hearing Loss and Deafness Panel integrate the detection of nuclear (e.g., *GJB2*, *SLC26A4*, *KCNQ4*) and mitochondrial variants (e.g., *MT-RNR1*, *MT-TS1*), and copy-number variations, providing diagnostic yields of 40–60% in worldwide populations. These protocols allow the identification of molecular etiologies and inform risk prediction models for individuals exposed to noise, solvents, or ototoxic medications, particularly those harboring variants in oxidative-stress or mitochondrial genes that increase environmental susceptibility as described before [32,50,51,60,65,256,267].

In translational research contexts, genotype-guided audiological surveillance is increasingly being proposed as a cost-effective strategy for precision prevention. For instance, screening for the well characterized *MT-RNR1 m.1555A* > *G* or *POLG* variants identifies patients at high risk of aminoglycoside- or noise-induced injury, prompting early audiometric monitoring and treatment modification [45,53,109,120,204,205,305]. Integration of NGS panels into occupational and environmental health programs could therefore help identify individuals with increased ototoxic vulnerability before clinical onset, potentially informing strategies to reduce the risk of irreversible sensory damage. As databases expand and variant interpretation algorithms are refined, these predictive models will underpin personalized hearing preservation frameworks that integrate genomic profiling, exposure assessment, and longitudinal audiological follow-up, marking a shift from reactive management to individualized auditory risk mitigation [120,123,311].

### 5.2. Gene Therapy and Epigenetic Approaches

Recent preclinical studies using mouse models in inner ear gene therapy have demonstrated that AAV-mediated restoration of *GJB2* and *OTOF* expression can effectively rescue hearing function in preclinical models of hereditary deafness [79,312,313,314]. In *Gjb2*-deficient mice, AAV vectors engineered to express wild-type *Gjb2* restored gap junction integrity and normalized intercellular ionic exchange, leading to partial recovery of auditory brainstem responses (ABRs) after a single round of local delivery [43,315,316,317]. More recently, base-editing AAV systems, such as SaCas9-NNG-ABE8e, have successfully corrected the dominant-negative *GJB2 R75W* mutation in vivo, repairing fragmented gap junction plaques and re-establishing physiological connectivity within the organ of Corti of transgenic mice [78]. These studies indicate that precise genomic repair or compensatory replacement of *GJB2* can reverse conductive pathway dysfunction and prevent the progressive degeneration that characterizes connexin-linked deafness.

Parallel breakthroughs have been achieved with AAV-mediated supplementation of *OTOF*. In *Otof*-knockout mice, AAV1 and AAV9 serotypes delivering full-length *Otof* restored exocytotic machinery and synaptic vesicle release, thereby reinstating ABRs and near-normal auditory thresholds sustained over several months [202,318,319,320]. Early human pilot trials (NCT05788536; ChiCTR2200063181) in children with bi-allelic *OTOF* mutations have reported functional hearing improvement from profound deafness to 50–70 dB HL on average, reflecting landmark translational progress with several children exhibiting hearing restoration in daily life conditions [321,322]. The convergence of these findings suggests that AAV-based gene replacement and base-editing therapies show promise for stable, transduction-specific correction of cochlear deficits in preclinical models and early clinical studies, offering a potential therapeutic platform for otherwise irreversible forms of genetic hearing loss.

Epigenetic modulation has emerged as a promising novel approach in the prevention of environmentally and drug-induced hearing loss, particularly using HDAC inhibitors and miRNA regulators that target oxidative stress and inflammatory pathways. HDAC inhibitors such as suberoylanilide hydroxamic acid (SAHA/vorinostat), belinostat, and panobinostat have shown robust protective effects in various preclinical models of hearing loss [83,238,323]. In an acute ototoxicity mouse model involving kanamycin and furosemide, systemic SAHA administration protected against hair cell loss and preserved hearing thresholds by restoring histone acetylation and activating cytoprotective genes involved in redox control and apoptosis inhibition [83].

In a NIHL model in CBA/J mice, SAHA helped prevent outer hair cell death and hearing impairment by modulating HDAC activity and histone acetylation [323]. Additionally, in a gentamicin-induced ototoxicity model in guinea pigs, the HDAC inhibitor sodium butyrate reduced HDAC1 expression, attenuated hair cell loss and improved auditory brainstem response thresholds [324]. Beyond histone regulation, HDAC inhibitors also act on non-histone substrates, modulating transcription factors such as NF-κB and Nrf2 to promote survival gene transcription while repressing inflammatory mediators [84,323].

Complementarily, miRNA-based approaches targeting oxidative-stress–responsive regulators, particularly miR-34a, which inhibits SIRT1, and miR-29b, which suppresses antioxidant enzymes, have demonstrated notable otoprotective potential. Down-regulation of these miRNAs or use of synthetic antagomirs restores mitochondrial homeostasis, reduces ROS accumulation, and preserves cochlear architecture in stress-exposed mice [252,253,325]. These findings emphasize that epigenetic therapeutics targeting chromatin acetylation or miRNA activity provide a mechanistically integrated approach to enhance the cochlea’s endogenous resilience to oxidative and inflammatory insults.

### 5.3. Antioxidant Interventions

Antioxidant-based interventions have shown protective effects against oxidative cochlear injury in preclinical models, particularly in contexts of environmental ototoxicity and occupational exposures [326]. Compounds such as N-acetylcysteine (NAC), CoQ10, and resveratrol, as well as *Nrf2*-activating agents, have consistently demonstrated protective efficacy in preclinical models of noise-, pollutant-, and drug-induced hearing loss, by restoring redox equilibrium and reducing mitochondrial dysfunction [52,53,292,327]. NAC provide intracellular glutathione, scavenges ROS, and improves vascular homeostasis, resulting in attenuated outer hair cell loss and preservation of auditory thresholds in rodents exposed to solvents and noise [328,329,330]. Similarly, CoQ10 enhances mitochondrial electron transport and prevents lipid peroxidation, while resveratrol exerts dual antioxidant and anti-inflammatory effects through *SIRT1* activation and Nrf2 upregulation, improving auditory recovery in animal models [292,326,331,332].

Recent research [93,284,333] also highlights pharmacological Nrf2 modulators, namely sulforaphane and bardoxolone-methyl, which induce endogenous antioxidant enzymes (HO-1, NQO1, GPX1), offering additional protection against solvent- or heavy metal-related inner ear toxicity. Another potential compound, mito-TEMPO, can penetrate the rat inner ear and, in a noise exposure model, attenuates cochlear oxidative stress, preserves mitochondrial integrity and biogenesis, and functionally protects against hearing loss, outer hair cell and synapse loss, as well as auditory nerve degeneration [209].

### 5.4. Occupational and Targeted Public Health Prevention

At the population level, translating these findings into occupational and public health prevention programs requires integration of large-scale datasets, with molecular biomarker information and systematic hearing surveillance. Implementation of biomarkers of oxidative stress, including malondialdehyde (MDA), 8-oxo-dG, and reduced/oxidized glutathione ratios, can enable early detection of redox imbalance in at-risk workers before clinical hearing deterioration [22,24,29,52,53,91,261].

Coupling such biomarkers with audiometric screening and individualized exposure profiles would optimize monitoring protocols within industries handling solvents, heavy metals, or high noise [254,275], in concordance with ethical standards. In parallel, regulatory enforcement focused on combined chemical–acoustic pollutant limits, engineering noise control, and antioxidant-rich nutritional education could form a comprehensive One-Health auditory protection strategy. Integration of preventive pharmacology with occupational health policy underscores a future vision of hearing loss prevention driven by molecular diagnostics and redox-modulating interventions [29,91,261].

## 6. Final Remarks and Perspectives

**Figure 2:**
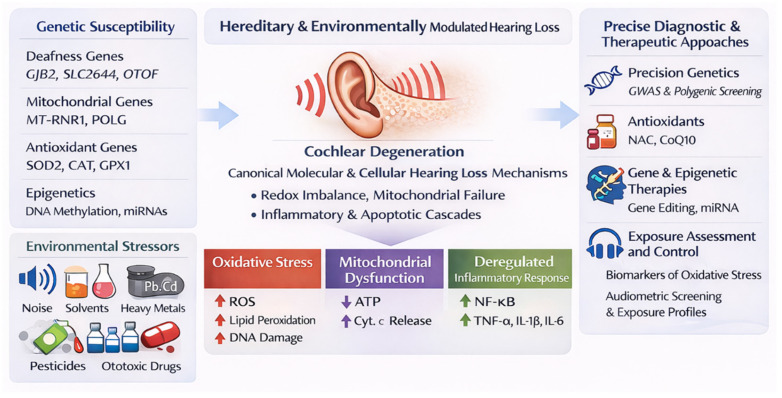
Gene–environment (noise and xenobiotics) interactions leading to cochlear degeneration and the perspectives on risk assessment and precise approaches for Hearing loss.

The intricate interplay between genetic susceptibility and environmental stressors defines the core of multifactorial hearing loss pathogenesis (Figure 2). Evidence from twin, animal, and large-scale genomic cohort studies consistently demonstrates that individuals carrying variants in oxidative stress, mitochondrial, and ionic transport genes (e.g., *SOD2*, *MT-RNR1*, *GJB2*, and *ATP2B2*) exhibit markedly increased vulnerability to auditory damage under noise, solvent, or metal exposure [29,41,50,51,55,57,73,87,256,260].

These gene–environment interactions operate through convergent mechanisms involving oxidative stress amplification, calcium dysregulation, mitochondrial impairment, and altered inflammatory signaling, thereby establishing a biological continuum between hereditary and acquired forms of sensorineural hearing loss [52,53,55,174]. Environmental ototoxins further exacerbate these processes by activating inflammatory and apoptotic cascades, such as the NF-κB or the caspase-dependent pathways, thereby accelerating hair-cell attrition and spiral ganglion degeneration [103,261,280,281].

As emphasized by recent environmental–genomics reviews, auditory susceptibility represents a dynamic interface between the genome and exposome, where the cumulative lifetime burden of pollutants and acoustic trauma reveals the limits of cochlear resilience [4,7,24,28,29,55]. In this context, emerging translational advancements in auditory precision medicine offer realistic paths toward prevention and repair. As referred to previously, promising gene replacement strategies using AAV vectors have already restored *GJB2*- and *OTOF*-related function in the murine model and in human clinical trials, while epigenetic therapies based on HDAC inhibitors and small-RNA modulators show potential to reverse oxidative transcriptional silencing [43,78,202,252,321,323]. Complementarily, antioxidant regimens targeting the Nrf2/HO-1 axis, including N-acetylcysteine, CoQ10, and resveratrol, have demonstrated efficacy in mitigating pollutant-induced cochlear damage in preclinical studies [93,209,284,327,328,332].

Yet, the long-term mitigation of hearing loss may depend as much on systems-level interventions as on molecular ones: integration of genetic screening into occupational surveillance, regulatory control of xenobiotic–acoustic exposures, and continuous monitoring of biological markers of oxidative stress could help translate discovery into public health protection [24,256,275,303,305]. Moreover, improved cellular and molecular/genetic resolution of cochlear and auditory nerve injury mechanisms is likely to strengthen cochlear implant practice by supporting etiology-informed stratification and counselling, refining outcome prediction, and enabling more individualized programming. This approach has already been adopted by several groups, who demonstrated associations between genetic background and post-implant oral language outcomes. Such insights are particularly relevant for distinguishing predominantly sensory (hair-cell) dysfunction from synaptopathic or spiral ganglion-predominant phenotypes, and for optimizing residual hearing preservation strategies in candidates for electric–acoustic stimulation [9,10,106,107,228,334,335]. Strengthening environmental genomics frameworks that integrate genotype data with exposure mapping will not only refine risk prediction but also guide policies aimed at mitigating what the WHO identifies as one of the fastest-growing global sensory health burdens.

The high costs of genetic testing and its implementation in public health programs remain significant challenges. Yet, the decreasing cost of next-generation sequencing and the development of targeted gene panels may make genetic testing more feasible in the near future. Coupling genetic testing with existing audiometric screening programs and exposure assessments could optimize monitoring protocols within industries handling solvents, heavy metals, or high noise [254,275]. Regulatory enforcement focused on combined xenobiotic–acoustic pollutant limits, engineering noise control, and antioxidant-rich nutritional education could form a comprehensive One Health auditory protection strategy.

## Figures and Tables

**Table 1:** Representative set of clinically and biologically relevant nonsyndromic hearing-loss genes, focusing gap junctions and intercellular signaling, synaptic transmission and mechanoelectrical transduction, and ion transport and endolymph homeostasis.

Gene	DFNA/DFNB Type (HHL)	Function in Hair Cells (Concise)	Association with Progressive Hearing Loss	Relevant References
***GJB2***	DFNB1	Encodes connexin 26; forms gap junctions in supporting cells and stria vascularis, important for K^+^ recycling	Variable; often congenital, may be stable or progressive depending on variant	[49,112,113]
***GJB6***	DFNB1 (deletion with *GJB2*)	Connexin 30; interacts with Cx26 in cochlear gap junction networks	Can contribute to progressive loss when combined with *GJB2* alleles	[112]
***SLC26A4***	DFNB4	Pendrin: anion exchanger in cochlea and endolymph homeostasis; associated with enlarged vestibular aqueduct	Often progressive or fluctuating, especially with enlarged vestibular aqueduct	[46,114,115]
***OTOF***	DFNB9	Otoferlin: Ca^2+^-sensing synaptic protein essential for vesicle release at inner hair cells ribbon synapses (auditory synaptopathy)	Typically congenital severe-to-profound non-progressive, but phenotype can vary	[9,116,117,118]
***MYO7A***	DFNA11/DFNB2 (also USH1B)	Unconventional myosin involved in stereocilia organization and cargo transport in hair cells	Variable; can be progressive in some DFNA cases	[108,119,120]
***MYO15A***	DFNB3	Unconventional myosin (Myosin XVa) required for stereocilia elongation and bundle development	Usually congenital severe-to-profound; progression rare	[121,122,123]
***TMC1***	DFNA36/DFNB7/11	Transmembrane channel-like protein implicated in mechanoelectrical transduction in hair-cell stereocilia	Dominant mutations often cause progressive hearing loss; recessive cause congenital profound loss	[120,122,124]
***TECTA***	DFNA8/12, DFNB21	Alpha-tectorin: extracellular matrix of the tectorial membrane, crucial for coupling of stereocilia	Many DFNA8/12 alleles cause mid-frequency progressive loss; DFNB21 often congenital	[125,126]
***CDH23***	DFNB12/USH1D	Cadherin 23: tip-link component for mechanotransduction and stereocilia cohesion	Recessive mutations often congenital severe; some dominant alleles cause progressive loss	[127,128]
***PCDH15***	DFNB23/USH1F	Protocadherin 15: tip-link component working with CDH23 in mechanoelectrical transduction	Recessive forms usually congenital; dominant missense variants may be progressive	[85,129]
***STRC***	DFNB16	Stereocilin: required for stereociliary bundle cohesion and auditory perception	Typically non-progressive congenital moderate-to-severe; some reports of mild progression	[130,131]
***KCNQ4***	DFNA2	Voltage-gated K^+^ channel in OHCs contributing to repolarization and cell excitability	Usually progressive high-frequency hearing loss (adult-onset progressive)	[132,133]
***TMPRSS3***	DFNB8/10	Serine protease implicated in hair-cell survival and cochlear homeostasis	Phenotype variable; can be prelingual progressive or postlingual progressive	[109,134]
***OTOG***	DFNB18	Otogelin: acellular gel-like structures of the otolithic and tectorial membranes; structural support of hair bundles	Usually congenital non-progressive but phenotype heterogeneity exists	[135,136]
***OTOA***	DFNB22	Otoancorin: mediates attachment of the tectorial membrane to the cochlear sensory epithelium	Often congenital; progression uncommon	[137]
***PJVK***	DFNB59	Pejvakin: involved in auditory nerve/hair-cell oxidative stress response and sound-evoked adaptation	Usually auditory neuropathy with variable progression	[138]
***SLC17A8***	DFNA25	VGLUT3: vesicular glutamate transporter in inner hair cell synaptic transmission	Progressive postlingual hearing loss reported in some families	[139]
***ESPN***	DFNB36	Espin: actin-bundling protein essential for stereocilia length/shape	Usually congenital; progression uncommon	[140]
***POU4F3***	DFNA15	Transcription factor required for hair-cell differentiation and survival	Typically progressive postlingual sensorineural hearing loss	[141]
***COCH***	DFNA9	Cochlin: extracellular matrix protein of inner ear affecting inner ear homeostasis and proteostasis	Progressive sensorineural hearing loss with vestibular dysfunction (adult-onset progressive)	[142]
***WHRN***	DFNB31	Whirlin: scaffolding protein at stereocilia tips, important for elongation and mechanotransduction	Recessive forms congenital; dominant alleles may show progression	[143]
***LHFPL5***	DFNB67	TMHS/LHFPL5: component of the mechanoelectrical transduction complex at tip links	Usually congenital; progression rare	[144]
***LOXHD1***	DFNB77	Important for stereocilia membrane homeostasis; multipass membrane protein with role in mechanotransduction	Often progressive postlingual in some families	[109,145]

DFNA: Deafness Autosomal dominant; DFNB: Deafness Autosomal recessive; HHL: Hereditary Hearing Loss.

## Data Availability

No new data were created or analyzed in this study.

## Abbreviations

The following abbreviations are used in this manuscript:

AAV	Adeno-Associated Virus
ANSD	Auditory Neuropathy Spectrum Disorder
ARHL	Age-Related Hearing Loss
ATP	Adenosine Triphosphate
CAT	Catalase
Cx26	Connexin 26
DFNA	Deafness Autosomal dominant
DFNB	Deafness Autosomal recessive
EVA	Enlarged Vestibular Aqueduct
GPX1	Glutamate Metabotropic Receptor 7
GRM7	Glutamate Metabotropic Receptor 7
GSR	Glutathione Reductase
GST	Glutathione S-Transferase
GSTM1	Glutathione S-Transferase Mu 1
GJB2	Gap Junction Beta-2 Protein
GJB6	Gap Junction Beta-6 Protein
HC	Hair Cell
HHL	Hereditary Hearing Loss
IHC	Inner Hair Cell
IL-1β	Interleukin-1 Beta
IL-6	Interleukin-6
MT-RNR1	Mitochondrially Encoded 12S rRNA
NF-κB	Nuclear Factor Kappa-Light-Chain-Enhancer of Activated B Cells
Nrf2	Nuclear Factor Erythroid 2-Related Factor 2
OTOF	Otoferlin
OHCs	Outer hair cells
POLG	DNA Polymerase Gamma
ROS	Reactive Oxygen Species
SLC26A4	Solute Carrier Family 26 Member 4
SOD2	Superoxide Dismutase 2
SGNs	Spiral ganglion neurons
TMC1	Transmembrane Chanel-like 1
TNF-α	Tumor Necrosis Factor Alpha
